# Percutaneous plasma laser disc coagulation and navigable ablation decompression in the treatment of cervical disc herniation: a single center experience

**DOI:** 10.3906/sag-1805-191

**Published:** 2019-02-11

**Authors:** Ayşegül CEYLAN, İbrahim AŞIK

**Affiliations:** 1 Department of Anesthesiology and Reanimation, Gülhane Education and Research Hospital, Ankara Turkey; 2 Department of Anesthesiology and Reanimation, Ankara University Faculty of Medicine Hospital, Ankara Turkey

**Keywords:** Navigable ablation, intradiscal decompression, percutaneous disc coagulation therapy, cervical herniation

## Abstract

**Background/aim:**

We aimed to compare the effectivity of percutaneous disc coagulation therapy (PDCT) and navigable ablation decompression treatment (L-DISQ) in patients who were diagnosed with cervical disc herniation.

**Materials and methods:**

Visual analog scale (VAS) and Neck Pain Index (NPI) scores were recorded initially and at the 1st, 3rd, 6th, and 12th months after the procedures. Patient Satisfaction Scale (PSS) scores were recorded 12 months after the procedures.

**Results:**

Mean VAS scores were 7.55 and 3.1 points in the PDCT group and 7.6 and 3.00 points in the L-DISQ group; mean NPI scores were 34.2 and 20.75 points in the PDCT group and and 34.1 and 20.4 points in the L-DISQ group initially and at the 12th month. When compared between months, there was a significant decrease in time-dependent VAS and NPI scores in both PDCT and L-DISQ groups (P = 0.001). Some complications included esophageal, vascular, and neural injuries; hoarseness; Horner syndrome; infections; dural puncture; and muscle spasm. The only difference between groups was the rate of cervical spasm within 1 month after the procedure: 75% in the PDCT group and 15% in the L-DISQ group.

**Conclusion:**

The diameter of the canal of the cervical vertebrae is narrower than of the lumbar and thoracic regions; therefore, the smaller part of the disc may be sufficient to create clinical signs. The response to decompression therapies is faster in the case of cervical percutaneous procedures that are performed correctly. Proper patient selection and practitioner’s experience are important in the treatment success.

## 1. Introduction

The most common cause of cervical pain and/or radiculopathy is the mechanical compression of the roots due to such degenerative spinal pathologies as cervical disc herniation (CDH) and spondylosis (1). When compared to the lumbar region, the diameter of the spinal canal and neural foramens are narrower in the cervical spine. Accordingly, although smaller in size and less protrusive, a cervical disc herniation has a higher risk of developing radicular symptoms than a lumbar herniation (2). Functionally, cervical vertebrae are more mobile and have a wider range of motion than the lumbosacral ones. Therefore, pain and limitation of motion in the cervical region lead to considerable discomfort (3). The most common radiculopathic finding is at the C7 nerve root location, followed by C6, C8, and C5 in descending order of frequency (4).

The choice of treatment for CDH is still controversial since the advantages of surgical treatment methods against nonsurgical ones have not yet been clearly defined (5). Interest in percutaneous procedures has increased due to the lesser threat of secondary tissue damage, operability with local anesthesia, avoidance of the risks associated with general anesthesia, and earlier discharge. As a result, interest in minimally invasive percutaneous methods for the treatment of CDH has increased, in which the nucleus pulposus is removed mechanically or through the application of energy (6,7).

Some of the complications are esophageal, vascular, or neural injuries; hoarseness; Horner syndrome; infections; leakage of cerebrospinal fluid; neurological damage; increase in pain; muscle spasm; and retrosternal and retropharyngeal pain (8–10).

There are some nonsurgical interventions for the treatment of CDH that usually aim to directly cauterize the discal parts, making use of various devices that approach anteriorly to the cervical disc (11,12). There is a need for comparative studies analyzing the results and long-term effects of these methods. There have been no publications investigating the performance of percutaneous disc coagulation therapy (PDCT) on the cervical region and only one study of cervical intradiscal navigable ablation decompression treatment (L-DISQ). To the best of our knowledge, this is the first study to evaluate the use of PDCT in the cervical region. The aim of this study is to compare the effectivity of percutaneous PDCT and L-DISQ ablation decompression treatments in patients diagnosed with CDH.

## 2. Materials and methods

Approval for the study was obtained from our institutional ethics committee. Data on patients who underwent treatment for pain in the posterior neck and in the superior extremities using L-DISQ and PDCT and/or with radiculopathy due to a CDH in the Pain Clinic between 2013 and 2017 was evaluated retrospectively.

### 2.1. Patient selection

From a total number of 105 patients with neck pain who were diagnosed with CDH by MRI between 2013 and 2017, 48 were treated with percutaneous decompression. Forty patients who were able to comply with the 12-month follow-up period were included in the study.

Inclusion criteria were as follows: Patients aged between 18 and 65 years old in the ASA I–II risk groups with with pain in the posterior neck and superior extremities and/or radiculopathy due to CDH were included in the study. All patients had cervical and upper extremity pain in most cases spreading to the scapular or occipital regions. None of the patients had only shoulder or neck pain. 

Exclusion criteria were as follows: Cervical myelopathy, cervical bone malformation leading to symptoms, upper motor neuron lesion findings with a history of cervical spinal surgery, extruded disc herniation, accompanying shoulder joint problems, symptom-related psychological discomfort, hematologic disorders, polyneuropathy, spinal cord stenosis, segmental instability or spondylolisthesis at the target level, infections, neoplastic diseases, and metabolic bone diseases.

The patients’ demographic data, visual analog scale (VAS) scores, and Neck Pain Index (NPI) scores were recorded initially and in the 1st, 3rd, 6th, and 12th months following the procedures. The Patient Satisfaction Scale (PSS) scores and cervical spasm rates were recorded in the 12th month following the procedures.

### 2.2. Used devices 

The PDCT system (Plasma D30, Jeisys Corporation, Seoul, South Korea) and L-DISQ (U&I Co. Uijeongbu, Korea) were used.

### 2.3. Procedure 

The same position and the same C-arm handling procedures were used in both patient groups. As two different devices were used, entry cannulas with different diameters, 1.2 mm and 0.8 mm, were used in the L-DISQ and PDCT procedures, respectively. 

In order to reduce the risk of infection and for sedation, 1 g of cefazolin and 2 mg of midazolam were injected intravenously (IV) prior the procedures. Intense sedation was avoided to allow full monitoring throughout the procedure. Patients were calm and able to speak to the practitioner when necessary. Blood pressure, heart rate, electrocardiogram, and oxygen saturation were monitored. While in a supine position, a thin roll was placed under the shoulders of the patient to allow a slight neck extension, and the shoulders were stabilized to allow better visualization of the lower cervical discs if needed. The neck was prepared and covered with sterile surgical cloths. First of all, all patients were injected with a local anesthetic of 2 mL of prilocaine into the skin and subcutaneous tissues. Deep injection was avoided during subcutaneous injection of the local anesthetic. This helped us to prevent development of side effects related to the local anesthetic agent such as Horner syndrome due to the proximity to the cervical sympathetic chain, bradycardia due to the effect on the carotid glomus, and bitonal sound or hoarseness due to the effect on inferior laryngeal nerve. An additional 0.5 µg/kg of fentanyl was administered intravenously in the case of pain until the disc was reached.

The port cannula was placed through the midline into the right side via an anterolateral approach. Since the esophagus is located on the left of the midline, a right-sided approach seems relatively safer, especially at lower cervical levels. Tight pressure was applied to the space between the trachea and the medial border of the sternocleidomastoid muscle in order to remove the trachea from the midline in a controlled manner. Bleeding/coagulation tests and anticoagulant drug use were questioned in all patients before the procedure. The carotid artery was sensed by the middle and index fingers to determine the entry point while preventing possible major vascular injury and hematoma. As the diameter of the cannula is small, tampon pressure was planned in case of hematoma. A possible headache due to dural injury was planned to be treated by daily abundant fluid intake, additional caffeine-containing painkiller agent, IV hydration, and epidural blood patch in the event that the headache did not cease after 1 week of treatment.

The cannula was moved obliquely into the intervertebral disc area 3–4 cm away from the midline at the index level. The C-arm was placed to obtain a lateral view of the surgical field, and then the cannula and trunk were advanced into the disc under fluoroscopic guidance. If both the anteroposterior and lateral views were located in the target area, we defined the needle position as “right”, and otherwise as “wrong”. The tip was retracted and retested in the event of such complaints as electric shock-like pain or muscle contractions. The pain resulting from a short test of electrical stimulation can prevent possible neural tissue damage. For both methods, the total duration of ablation after test stimulation was 100–150 s. 

All patients were prescribed antibiotics to use for 7–10 days and to take an antiinflammatory drug or paracetamol in the case of pain, along with a hard collar to be worn for 2 weeks during the day. Patients were informed not to drive for at least 72 h. 

No patient required additional invasive pain intervention after the procedure.

### 2.4. Statistical analysis 

The data analyses were carried out using SPSS 15 for Windows. Descriptive statistics were expressed as mean ± standard deviation for variables with normal distribution, median (min–max) for variables with abnormal distribution, and number of cases and percentage (%) for nominal variables. Within the groups, the significance of the difference in median values and in the median values between times was assessed with a Friedman test. If present, multiple intertime comparisons of differences were evaluated using appropriate post hoc tests. Between the groups, the significance of the difference in terms of average values was evaluated by two related sample tests, and in terms of median values by a Wilcoxon test.

P < 0.05 was considered to be statistically significant, and a recovery rate of over 50% was considered successful.

## 3. Results

Forty patients were included in the study, of which 47.5% were female and 52.5% were male, with a mean age of 53 years. Lesions were predominantly at the C4–C5 and C5–C6 distances, as summarized in Table 1. 

**Table 1:** Demographic data.

PDCT	n	%	L-DISQ, n	%
Sex				
Male	9	42.9	12	57.1
Female	11	57.9	8	42.1
Level				
C3–C4, C4–C5	5	25.0	3	15.0
C4–C5, C5–C6	7	35.0	7	35.0
C5–C6	3	15.0	4	20.0
C5–C6, C6–C7	3	15.0	3	15.0
C4–C5	2	10.0	3	15.0
Cervical spasm				
Yes	13	65	4	20
No	7	35	16	80
PSS				
Very good	1	5.0	1	5.0
Good	14	70.0	16	80.0
Moderate	5	25.0	3	15.0

PSS: Patient Satisfaction Scale; PDCT: plasma laser disc coagulation.

The time-dependent VAS scores were found to be statistically significant for each treatment method (P = 0.001), as presented in Table 2. In a comparison of the PDCT and L-DISQ groups, the difference between the VAS scores in the 0th, 3rd, 6th, and 12th months was statistically insignificant, while the difference between the VAS scores in the 1st month was statistically significant (P = 0.033). In the PDCT group, the time-dependent VAS scores between the 1st and 3rd months were significant at P = 0.039 and between the 3rd and 12th months were significant at P = 0.012. In the L-DISQ group, the time-dependent VAS scores between the 0th and 1st, 0th and 3rd, 0th and 6th, and 0th and 12th months were P = 0.0019, and between the 3rd and 12th and 6th and 12th months were P = 0.008, and thus statistically significant (Table 2).

**Table 2:** VAS score data.

Comparison of VAS scores according to method, PDCT vs. L-DISQ			
Time	VAS PDCT	VAS L-DISQ	P^1^
0th month	7.55 ± 0.61	7.6 ± 0.68	0.88
1st month	3.55 ± 0.51	3.15 ± 0.59	0.033*
3rd month	3.6 ± 0.6	3.5 ± 0.51	0.643
6th month	3.3 ± 0.57	3.35 ± 0.59	0.768
12th month	3.1 ± 0.55	3 ± 0.56	0.568
Comparison of time-dependent VAS scores			
Time	VAS PDCT / P^2^	VAS L-DISQ / P^2^	
0–1 months	0.001*	0.001*	
0–3 months	0.001*	0.001*	
0–6 months	0.001*	0.001*	
0–12 months	0.001*	0.001*	
1–3 months	0.763	0.052	
1–6 months	0.225	0.157	
1–12 months	0.039*	0.317	
3–6 months	0.083	0.366	
3–12 months	0.012*	0.008*	
6–12 months	0.206	0.008*	

VAS: Visual analog scale. *Significant for P < 0.05, P1: Wilcoxon Mann–Whitney U; P2: Friedman test.

The time-dependent NPI scores were found to be statistically significant for each treatment (P = 0.001). Time-dependent NPI scores are shown in Table 3. In a comparison of the PDCT and L-DISQ groups, the differences between the NPI scores in the 0th, 1st, 3rd, 6th, and 12th months were statistically insignificant. In the PDCT group, the differences in the time-dependent NPI scores between month 0 and the other months were statistically significant at P = 0.001. The differences between the 1st and 3rd, 1st and 6th, and 1st and 12th months were also statistically significant at P = 0.001, 0.011, and 0.006. In the L-DISQ group, the time-dependent NPI scores between month 0 and all the other months, and also between the 1st and 6th months, were statistically significant at P = 0.001, while the scores between the 1st and 3rd months were also statistically significant at P = 0.009 (Table 3).

**Table 3:** NPI score data.

NPI scores according to method, PDCT vs. L-DISQ			
Time	NPI PDCT	NPI L-DISQ	P^1^
0 month	34.2 ± 1.64	34.1 ± 1.48	0.89
1st month	22.85 ± 2.25	21.6 ± 1.93	0.095
3rd month	20.7 ± 1.3	20.4 ± 1.05	0.238
6th month	21.1 ± 1.37	20.65 ± 1.09	0.427
12th month	20.75 ± 1.37	20.4 ± 0.1	0.273
Comparison of time-dependent NPI scores			
Time	NPI PDCT / P	NPI L-DISQ / P	
0–1 months	0.001*	0.001*	
0–3 months	0.001*	0.001*	
0–6 months	0.001*	0.001*	
0–12 months	0.001*	0.001*	
1–3 months	0.001*	0.009*	
1–6 months	0.011*	0.001*	
1–12 months	0.006*	0.077	
3–6 months	0.269	0.349	
3–12 months	1.000	1.000	
6–12 months	0.287	0.479	

NPI: Neck Pain Index. *Significant for P < 0.05, P1: Wilcoxon Mann–Whitney U; P2:Friedman test.

Among the PDCT patients, one patient rated the PSS as very good, while 14 patients and 5 patients rated it as good and moderate, respectively. Among the L-DISQ patients, 1, 16, and 3 rated the PSS as very good, good, and moderate. Of the 40 patients included, 2, 30, and 8 rated the PSS at the 12th month as very good, good, and moderate, respectively. Overall, 80% of the patients evaluated the procedure as very good or good according to the PSS (Table 1).

## 4. Discussion

In recent years, less invasive procedures have been developed for the treatment of patients with CDH, including those that use mechanical, chemical, and thermal methods to compress the discs (13,14). 

Although authors have previously addressed lumbar interventions in the literature, the present study discusses cervical interventions. 

During the follow-up year, a statistically significant reduction was noted in both VAS and NPI index scores in the PDCT and L-DISQ groups. The neck pain felt during the procedure and the risk of postoperative muscular spasm was reduced. Cervical spasm developed in 13 and did not develop in 7 of the PDCT patients, and among the L-DISQ patients, cervical spasm developed in 4 and did not develop in 16. The patients who experienced cervical spasms benefited from such short-term palliative treatments as supportive physical therapy, hot compress, and analgesics (Table 1).

PDCT works by using a laser source called a plasma light, and produced plasma rays condense at the end of the dome-shaped probe and do not move forward. A fire sphere with a diameter of 4 mm is created around the coagulation area and all energy is concentrated at the end of the fiber. The PDCT system is a type of therapeutic system for disc coagulation, evaporation, and decompression with plasma. Most lasers use a power of 900 J in a pulsatile mode at cervical distances (15). The area within 4 mm around the tip on the fiber is lower than 40 °C for safety. This is why adjusting and healthy tissues are protected from thermal elevations. The diameter of the plasma fiber optic is 0.4 mm. The basic principle is to create 3 lesions in the intradiscal region. During the process, the evaporated disc is checked for output by opening a three-way valve (16). The total surgical procedure takes 15 min.

Bellini et al. reported preliminary results of 19 patients with lumbar or cervical disc herniation treated with PDCT, defining the process according to quarterly evaluated VAS scores. No serious complications were reported, aside from mild muscular spasm and stomach complaints in 10 patients. Despite the fact that PDCT uses a modified plasma laser technology, we believe that the cervical muscular spasms were related directly to the high power effect of the applied energy (15,16).

L-DISQ is a device that approaches the herniated disc sensitively along the intradiscal pathway and vaporizes the herniated nucleus using plasma particles and bipolar radiofrequency plasma for decompression. This device has been replaced with a valid form called L-DISQ-C to enter the cervical intervertebral space (17). 

The navigable electrode end of the L-DISQ is twisted by turning the control wheel and can penetrate completely into the discal hernia. This feature provides direct access to the herniated part of the disc to be ablated and reduces the amount of ablated discal tissue and fibrous damage to the outer ring (18). 

Since the distance between two electrodes at the end of L-DISQ is 0.2 mm, a nerve root farther from the electrode tip than 0.2 mm is theoretically protected from electrical damage. During the procedure, electrical current passes through the other electrode instead of the nerve root (17). Additionally, the thin outer membrane ring is a good barrier for electrical current and theoretically reduces nerve damage due to bipolar electrical coupling (13). During controllable ablation, the highest recorded temperature is between 40 and 60 °C and the temperature decreases rapidly with distance (19). This is why adjusting and healthy tissues are protected from thermal elevations. Temperatures do not rise inside or on the surface of the disc, but do rise significantly around the probe tip. As a result, nerves and surrounding tissues are protected from the risk of thermal damage as long as the probe tip is within the disc (20). The total surgical procedure takes 15 min.

Lim et al., following interventions with L-DISQ in patients with extruded and protruded cervical disc herniation, came to conclusions that paralleled those of the present study (21). In the study by Kim (22), L-DISQ had technical properties that facilitated its use in extruded cervical disc hernias.

Unlike in our study, previous studies using L-DISQ in the treatment of cervical disc herniation included extruded patients, but we only treated patients with protruded discs. We believe that it is more suitable to access to the target tissue in protruding disc hernias with higher rates of success. For the placement of the electrode by navigation within the herniated part, the experience of the practitioner and proper patient selection are important factors affecting success. Precise access to the target is crucial for success. In the present study, we observed no indications that the electrode had been falsely placed.

In some of the patients in the present study, intradiscal treatment was applied at multiple levels, while all interventions in other studies were made at a single level. 

Although PDCT is used to reduce the problems associated with other laser applications, its probe cannot be seen in the disc through fluoroscopy, which can be considered a disadvantage (Figure). Since the entrance cannula of the PDCT is thinner than the L-DISQ cannula, intradiscal access is easier, despite the potential for bone surface problems. This feature provides significant benefit to the user. L-DISQ can be monitored under fluoroscopy, including its tip, which is an advantage for L-DISQ users. 

**Figure 1:**
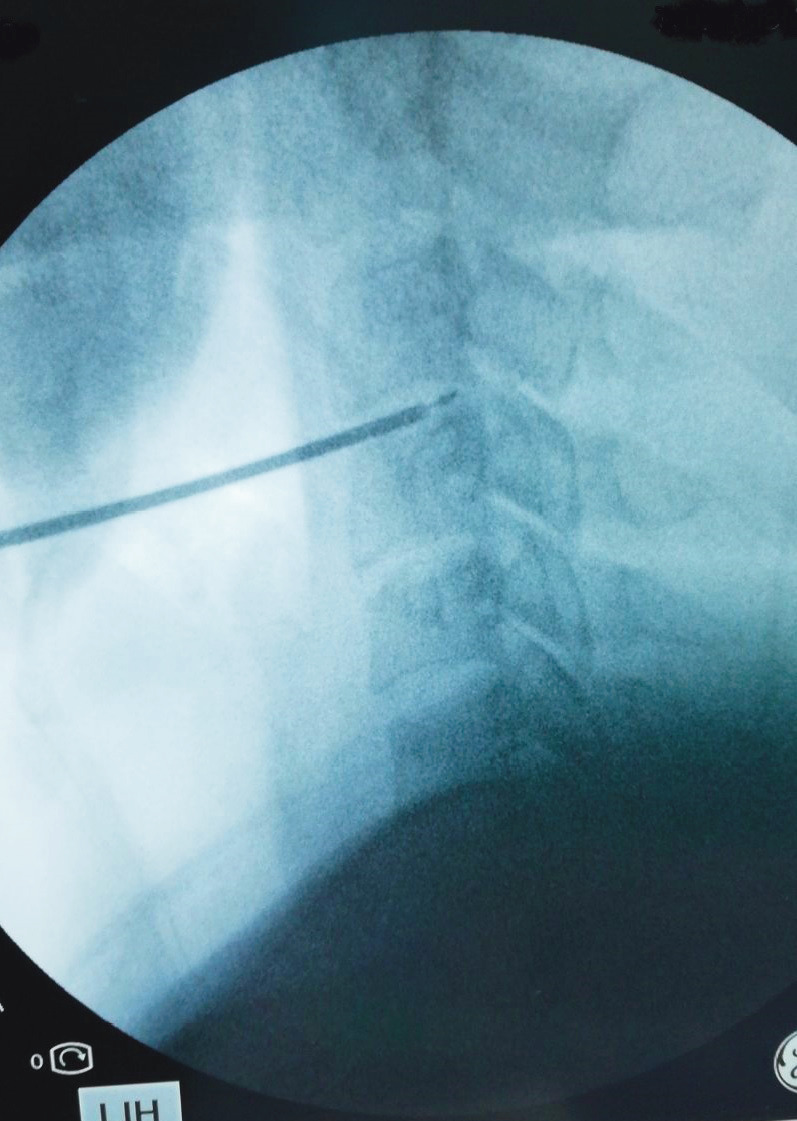
Although PDCT is used to reduce the problems associated with other laser applications, its probe cannot be seen in the disc through fluoroscopy.

In our study, we did not perform MRI routinely for follow-up after the procedure. The pain and functional status was determined by clinical anamnesis and physical examination. Bonaldi et al. (8) reported that clinical improvement was not always accompanied by MRI findings. Schellhas et al. (7) reported that pain did not always correlate with radiological findings. Similarly, Arana et al. (18) did not observe a significant correlation between MRI findings and pain and various clinical signs.

In conclusion, L-DISQ provided better pain control than PDCT in the early period while both methods were found to have similar effectivity in the long term. In the PDCT group, the higher VAS score values in the early period were thought to be associated with paracervical muscle spasms. The outcome of neither treatment method was superior to the other in terms of the outcomes of the two groups in treatment follow-up. The decreases in the VAS and NPI scores of the patients were statistically significantly. We believe that PDCT and L-DISQ can be considered safe and efficient procedures in patients with CDH.

Although NPI evaluates the functional improvements of CDH patients and includes some questions judging parameters such as social life, daily functionality, and sleep quality, some additional indexes and questionnaires supporting these parameters can be evaluated in further studies including larger patient populations.
